# Awareness Regarding an Infant’s Sleep Environment and Safe Sleep Practices Among Polish Caregivers: A Cross-Sectional Survey

**DOI:** 10.3390/jcm14124295

**Published:** 2025-06-17

**Authors:** Agata Michalska, Anna Zmyślna, Justyna Pogorzelska, Marta Mierzwa-Molenda, Justyna Mazur, Aleksandra Gładyś-Jakubczyk, Marek Żak

**Affiliations:** 1 Institute of Health Sciences, Collegium Medicum, Jan Kochanowski University, 25-369 Kielce, Poland; michalska.agata@ujk.edu.pl (A.M.); justyna.pogorzelska@ujk.edu.pl (J.P.); aleksandra.gladys-jakubczyk@ujk.edu.pl (A.G.-J.); mkzak@ujk.edu.pl (M.Ż.); 2 Neonatology Clinical Department, Provincial Hospital in Kielce, 25-736 Kielce, Poland; justy.mazur@gmail.com

**Keywords:** sudden infant death syndrome, SIDS, sleeping practice, prevention

## Abstract

**Background:** Effective prevention of SIDS/SUID requires a multidimensional approach. It is essential to provide targeted support for marginalized families, improve access to healthcare services, and implement policies aimed at reducing social and economic inequalities. The parallel effective way to reduce the risk of sudden unexpected infant death is through increased awareness of proper infant care. Despite the guidelines available in many countries, the risk of infant death from non-medical causes is still reported. The aim of the study was to assess awareness regarding an infant’s sleep environment and safe sleep practices among Polish caregivers. **Methods:** The survey was conducted among 451 mothers of infants under 18 months of age. The survey questionnaire was prepared, which consisted of single- and multiple-choice questions with closed and open answers assessing safe sleep practices. **Results:** Analysis of the survey responses showed that most mothers in the sample (88.9%) were aware that the supine position is the safest. According to the survey, 74.5% of respondents believed that infants should not sleep in the same bed as parents or siblings. In addition, 78.3% of those women stated that infants should sleep separately in their own bed. Despite this knowledge, almost 37.76% of female respondents still put their infants to sleep in their parents’ bed. As many as 98.4% of respondents knew that any items should not be placed in an infant’s crib. Despite this knowledge, a third of respondents still placed additional items in their baby’s crib. In the study 90.7% of women believed that breastfeeding should begin within the first hour after birth. **Conclusions:** Knowledge of safe sleep recommendations, including sleep environment, sleep position, and spatial organization, does not always translate into proper caregiving practices. Understanding caregivers’ motivations and how they perceive medical information is critical to effective prevention of infant sleep safety.

## 1. Introduction

Explained or unexplained sudden infant death is referred to as sudden unexpected infant death (SUID; explained or unexplained), sudden infant death syndrome (SIDS; unexplained), or sleep-related death (explained or unexplained) [1]. Recommendations for safe infant sleep are constantly adjusted to the latest medical knowledge. Despite numerous campaigns in many countries around the world, the occurrence of SIDS cases is still classified as a major cause of postnatal mortality in favor of safe sleep and progress in understanding risk factors. In order to reduce the risk of SIDS and SUID, many countries such as the United Kingdom, the United States, Ireland, Australia, Spain, and Norway have introduced standards of care for infants [2].

The most recent update took place in 2022 [1]. It was recommended that infants aged up to 1 year should be placed in supine position on a firm, non-inclined sleep surface and that infants should sleep in a separate bed, avoid overheating, and keep their heads uncovered. Additional recommendations for reducing the risk of sleep-related death include breastfeeding, pacifier use, routine immunizations, and avoiding nicotine exposure and alcohol use. Tummy time, while the infant is awake as a method of brachycephaly prevention and motor development facilitation, should be implemented (at least 15–30 min total daily by age 7 weeks). In Poland, the standards of ambulatory care for preterm babies (2022) [3] remain in effect. Polish guidelines are primarily based on AAP recommendations, which is largely due to the absence of national epidemiological data and comprehensive research. Nevertheless, it is essential to adapt these recommendations appropriately to the local sociocultural context in order to enhance their effectiveness and public acceptance [4].

These standards were developed by a team of experts from the Polish Neonatological Society, the country’s most important source of expert opinions in the field of neonatology. In the chapter regarding SIDS, recommendations are consistent with the global guidelines [1,5,6].

It is emphasized that the baby should sleep in its own crib. The child should not be placed to sleep in car seats, bouncers, carriers, or strollers. Based on these standards, medical professionals (doctors, midwives, nurses, physiotherapists) educate caregivers. This education most often takes place in prenatal classes, hospitals, clinics, or during home visits. However, statistics on sleep-related deaths confirm that despite the efforts, unsafe sleep practices are still being followed. The implementation of safe sleep guidelines by caregivers remains challenging. In Poland, there is no systematic collection of epidemiological data on SIDS. To date, no studies have been carried out that investigate safe infant sleep practices. To assess the extent of knowledge and beliefs regarding safe infant sleep practices that reduce the risk of SIDS, the authors conducted research among mothers of children up to 18 months of age. The aim of the study was to assess knowledge regarding infant’s sleep environment and safe sleep practices among Polish caregivers.

## 2. Materials and Methods

The survey involved 451 female participants, recruited between March 2024 and September 2024. The study population consisted of mothers caring for infants under 18 months of age. Participation in the survey was voluntary and took place during prophylactic medical visits or physiotherapy appointments at healthcare facilities. The research was conducted in Kielce, a regional city and the administrative capital of the świętokrzyskie voivodeship in south-central Poland. Participants completed a questionnaire and provided informed consent for the use of their data in research. They were free to withdraw from the study at any time without any legal or financial consequences. Completion of the questionnaire was considered as implicit consent to participate. The study was conducted anonymously, ensuring that no personally identifiable information was collected. The questionnaire was developed based on current authoritative recommendations regarding safe infant sleep. Items were carefully selected by experts following a thorough literature review and consultations with pediatricians, neonatologists, and physiotherapists who work directly with infants. The survey employed an exploratory design, encompassing various independent aspects of parental knowledge and practices. The primary aim was to capture a comprehensive spectrum of behaviors and beliefs related to safe sleep practices. Responses were provided anonymously and independently, without influence from others. Data analysis was conducted by individuals who were not involved in data collection, ensuring objectivity.

An original questionnaire was used in the study which was based on the AAP guidelines for safe sleep [1] and standards of ambulatory care for preterm babies [3]. It contained closed-ended questions assessing knowledge in the area of safe sleep as well as open-ended questions regarding safe sleep practices. The study was approvedby the Bioethics Committee of Jan Kochanowski University in Kielce, NR 50/2021.

## 3. Results

A total of 451 women participated in the study. The average age was just over 30 years (30.56, SD = 4.32, min 18, max 45). The largest group consisted of women over the age of 30. The vast majority had higher education (83.6%). About one-third represented medical professions. The characteristics of the study group are presented in Table 1.

The survey included eight questions regarding safe sleep. Each correct answer was awarded 1 point, while incorrect answers received 0 points. The maximum achievable score was 8. The average knowledge score on safe sleep was 5.6 (SD = 1.44), indicating a good level of knowledge. Less than 10% of respondents scored below 4 points. Among the participants, 21.3% scored 5 points, 24.6% scored 6 points, 25.1% scored 7 points, and 6.7% scored 8 points. Neither the education level nor the age of the mothers significantly affected their knowledge. Women in medical professions had higher scores (5.93; 1.42 SD vs. 5.52; 1.44 SD; *p* = 0.007). Parity also significantly influenced knowledge levels (one child vs. two or more children, 5.77; 1.38 SD vs. 5.40; 1.51 SD).

The study assessed the frequency of correct and incorrect responses to individual survey questions concerning safe sleep knowledge. The highest percentage of incorrect responses was related to side sleeping, with 60% of respondents considering it a safe position. One in four mothers believed that co-sleeping with parents or siblings was safe for infants. Almost all respondents agreed that additional objects should not be placed in the infant’s crib. A significant majority of women believed that breastfeeding should begin within the first hour after birth and should not be on demand. According to 96.5% of respondents, breastfeeding mothers should follow an elimination diet, which involves removing certain foods from their diet to identify and avoid potential allergens that might affect the infant. Only 43.9% of respondents were aware that pacifier use can reduce the risk of sudden infant death syndrome (SIDS). However, 85.8% correctly recognized that using a pacifier during breastfeeding initiation may disrupt proper sucking mechanisms and contribute to lactation issues (Table 2).

The study also examined mothers’ choices regarding their infants’ sleep locations. Over half of the respondents (59.6%) placed their child to sleep in only one location, 22.2% used two locations (most commonly a crib and the parents’ bed), and 10.9% used three locations (most commonly a crib, parents’ bed, and stroller). Other respondents used four or more locations. Correlation analysis showed no statistically significant relationship between the child’s age and the number of sleep locations (rho = −0.06, *p* = 0.183, *n*= 451).

Regardless of the child’s age, the most frequently chosen sleep location was a crib (76.8%). Nearly half of the infants slept in the parents’ bed (47.2%), while 15.1% slept lying on a parent. A stroller was used in 16.7% of cases. Less than 5% of respondents used a baby nest (4.7%), cradle (4.2%), or sleeping basket (3.6%). In each age group, the most frequently indicated place for a child’s sleep was a crib, followed by the parents’ bed, and thirdly, the stroller. In the studied group, older children showed a tendency to abandon sleeping in the crib and stroller in favor of sleeping in the same bed with their parents. The highest percentage of children falling asleep in their parents’ bed was recorded in the group of 8–12-month-olds (Table 3).

The compared age groups differed significantly only in terms of sleeping in the parents’ bed (χ^2^ = 18.56, df = 3, *p* < 0.001, Vc = 0.20) and sleeping in the crib (χ^2^ = 9.06, df = 3, *p* < 0.029, Vc = 0.14). Children up to 3 months old slept significantly more often in a crib, whereas they slept significantly less often in the parents’ bed compared to older children. No statistically significant differences were observed between age groups in terms of other sleeping places (*p* > 0.05).

Among the surveyed mothers, 353/451 confirmed that infants should sleep separately in their own cribs. However, nearly one-third (133/353; 37.76%) still placed their child in the parents’ bed. A total of 17% respondents (60/353) stated that their children always sleep with the parents, and this is their only sleep location.

In addition to sleep locations, the study analyzed the sleeping positions of infants. The majority of mothers placed their child to sleep on their back, more than one-third placed them on their side, and about 10% placed them on their stomach (Table 4).

Among the surveyed mothers, 311 out of 451 stated the prone sleeping position could be dangerous for infants. However, 8.4% (26/311) of them still reported placing their child in this position for sleep. To determine whether the child’s age was a significant factor differentiating sleeping positions, a non-parametric chi-square independence test (χ^2^) was conducted. Significant differences between groups were found only in the frequency of placing infants on their back (χ^2^ = 22.37, df = 3, *p* < 0.001, Vc = 0.22). Infants aged 13 months and older were significantly less likely to be placed on their back compared to younger infants (Table 5). No statistically significant differences between age groups were observed for other sleep positions (*p* > 0.05).

In the studied group, one in three mothers (30.8%) placed an object in the infant’s crib. This percentage was comparable across all age groups. The most commonly placed objects were crib bumpers (39.6%), pillows (38.8%), and cloth diapers (37.4%), while stuffed toys were less common (12.9%). More than half of the mothers (60.3%) reported that their child used a pacifier, with similar rates across all age groups. Among the surveyed mothers, 73% breastfed their infants, and 96% followed the recommended vaccination schedule.

## 4. Discussion

Sudden Infant Death Syndrome (SIDS) was first described in 1969. It is a complex and multifactorial phenomenon that leads to the unexpected death of an infant within the first year of life. Sudden Infant Death Syndrome (SIDS) occurs during sleep and remains unexplained even after a thorough autopsy, medical history review, and scene investigation. Therefore, public health campaigns aimed at promoting safe sleep practices have played a crucial role in reducing SIDS incidence globally. Initiatives such as “Back to Sleep” in the UK and US, “Sleep Safe, My Baby” in Australia, and culturally adapted interventions in New Zealand have significantly lowered SIDS mortality. These efforts emphasize evidence-based recommendations, including placing infants on their backs to sleep, avoiding soft bedding and overheating, and reducing exposure to tobacco smoke. Although Poland has not launched a nationwide media campaign, healthcare professionals convey safe sleep guidelines in accordance with international standards [1,7,8,9,10,11,12].

Despite such a broad health policy and education implemented in previous years, statistics still show that the problem persists. According to data from 2022, in the United States, 3700 children died due to Sudden Unexpected Infant Death (SUID), of which 1529 deaths were attributed to SIDS [13]. Based on Eurostat data, the number of infant deaths in Poland in 2020, 2021, and 2022 was 1270, 1306, and 1176, respectively, with SIDS accounting for 15, 19, and 19 deaths in those same years [14]. In Poland, there is no dedicated system for collecting epidemiological data on SIDS [3].

Over the past 20 years, the incidence of SIDS in the USA has decreased by more than 50%, partly due to the “Back to Sleep” campaign launched by the American Academy of Pediatrics (AAP) in 1994, which raised awareness about a safe sleep environment [15].

The etiology of SIDS remains unclear. The causes of sudden infant death syndrome are linked to genetic, environmental, and social factors. A widely cited “triple-risk” model includes factors such as a critical period in infant development, the infant’s vulnerability, and external risk factors. Despite extensive research, the primary mechanism of SIDS has not been established. However, several risk factors are known, including complications during pregnancy, prematurity, low birth weight, male sex, maternal substance use (including smoking), improper sleep position (prone or side sleeping position), sleeping on soft surfaces, covering the infant’s head, and overheating. The highest incidence of SIDS is observed in the second, third, and fourth months of life [16,17,18,19,20,21,22].

To enhance sleep safety, the American Academy of Pediatrics recommends placing infants on their back, using a firm, flat sleep surface, room-sharing without bed-sharing, avoiding soft bedding and overheating. Additional recommendations include breastfeeding, pacifier use, routine vaccinations, and avoiding exposure to nicotine, alcohol, marijuana, opioids, and other drugs [1].

One of the most well-known risk factors for SIDS is infant sleep position. According to Adams et al. [8], prone or side sleeping position nearly doubles the risk of SIDS [21]. Analysis of survey responses indicates that most mothers (88.9%) are aware that the back position is the safest. Only 12% place their infants on their stomachs to sleep, and nearly half of them are aware that this position is not recommended by specialists. In contrast, the side position is not commonly identified as potentially dangerous. More than half of respondents considered it safe, and one-third of infants sleep in this position. In a study conducted by Alahmadi et al. [22], only 63.2% of infants were placed to sleep on their back, with 68.9% of those under six months of age and 53.2% of those over six months of age. Some researchers suggest that the proportion of infants sleeping on their stomachs increases with age. It was found that at one month old, 12% of infants were placed in prone sleeping position, and by three months, this percentage increased to 20% [23,24]. A similar trend was observed in Swedish studies, where the frequency of prone sleeping was 1.3% in infants up to three months old, 5.6% in those aged three to five months, and 5.3% in those aged six to eight months [25].

In our study, this percentage ranged between 12–13.6% during the first year of life and did not show an increasing trend. The proportion of infants sleeping on their side also increased with age, from 14.3% in the first three months to 31.1% between six and eight months. At six months, 27.3% of infants slept on their sides [25]. In our study, this percentage was higher, ranging between 30.5–42% during the first year of life.

Apart from sleep position, the main recommendations for safe sleep include the sleep environment and the organization of space. A firm mattress is recommended. Infants should not be placed to sleep on inclined sleep products such as hammocks, baby nests and pods, compact bassinets without stands or legs, sofas, travel bassinets, infant tents, car seats, or bouncers [22,26]. Infants should sleep in their own crib. Bed-sharing with an infant younger than four months has been associated with an increased risk of SIDS. Sharing a bed with a person whose alertness or ability to wake up is impaired due to fatigue, sedatives, or substance use can also contribute to sudden infant death. For this reason, more researchers recommend that infants sleep in the same room as their parents but in a separate crib [19,27].

According to our study, 74.5% of respondents believed that infants should not sleep in the same bed as parents or siblings. Additionally, 78.3% of women stated that infants should sleep separately in their own crib. Despite this knowledge, nearly 37.76% of respondents still placed their infants in the parents’ bed for sleep. In a study by Möllborg et al. [28], 65.7% of infants slept in a separate crib in their parents’ room. Similar results were found in a study conducted in New Zealand, where 61.3% of infants usually slept in their parents’ room but in their own crib [29]. Among Thai infants, the rate of room-sharing (but not bed-sharing) was 39.3% [30]. Meanwhile, a study by Rohan et al. [31] found that 68.8% of Malaysian parents always or frequently put their infants to sleep in their own crib. An Australian study conducted on 174 women reported that 33% of participants practiced bed-sharing with their newborns aged 0–1 month [32]. Except for the Thai study, these findings align with the results of our study. Some researchers suggest that bed-sharing with an infant correlates with an increased likelihood of breastfeeding [33]. Osberg et al. [34] found that 43% of three-month-old infants slept in their parents’ bed. Among them, 42% slept in a baby nest, while another 42% slept in close contact with a parent. Bed-sharing was most commonly associated with breastfeeding.

Additional objects in an infant’s crib can increase the risk of SIDS up to threefold [1]. As many as 98.4% of respondents knew that items such as pillows, toys, diapers, and other objects should not be placed in an infant’s crib. Despite this knowledge, one-third of respondents still placed extra objects in their child’s crib. A study by Nelson et al. [35], which examined childcare practices across 21 sites in 17 countries, found that families in Hungary (0%), Scotland (4%), Canada (8%), and New Zealand (9%) reported the lowest rates of pillow use, while Chinese groups (Hong Kong 80%, Beijing 95%, and Chongqing 95%) reported the highest rates. Coll et al. emphasized that caregivers’ ethnic background significantly influenced the use of soft objects or bedding, particularly pillows [24].

According to research by Alahmadi et al. [22], breastfeeding reduces the risk of SIDS by half. The American Academy of Pediatrics recommends exclusive breastfeeding or feeding by expressed breast milk for approximately first 6 months of life, (and beyond—continue breastfeeding with complementary foods for at least 2 years). Premature infants and those with low birth weight, who are at higher risk of SIDS, should especially be breastfed. It is important to emphasize the benefits of breast milk in developing the infant gut microbiome and reducing the risk of infectious diseases during the first year of life. Collaboration with families of preterm infants is crucial to ensure intensive support during prolonged hospitalization in neonatal intensive care units [3,19,23,27].

In the study group, 73% of mothers breastfed, while 90.7% of women believed that breastfeeding should begin within the first hour after birth. Data analysis conducted by Thompson et al. [36] showed that breastfeeding must last for at least two months to provide protection against SIDS, with the risk decreasing by nearly half. The longer breastfeeding was maintained, the greater the protective effect against SIDS. An Iranian study found that parental education levels were significantly associated with breastfeeding rates [37]. Bailey et al. [32] and Cole et al. [38] highlighted those factors predicting breastfeeding included maternal education, mode of delivery, maternal age, marital status, smoking status, formula supplementation in the hospital, neonatal unit admission, pacifier use, lack of confidence, lack of knowledge, and conflicting advice.

To reduce the risk of SIDS, researchers recommend vaccinating infants according to the current Immunization Schedule. Although no direct causal connection between vaccinations and SIDS has been proven, many studies suggest that vaccinations help reduce infection risk and improve overall infant health [3,19,26]. Scientific research also confirms that pacifier use during sleep has a protective and soothing effect on infants, reducing the risk of SIDS. Using a pacifier may help maintain airway patency and regulate the autonomic nervous system during sleep [3,25]. In the study group, more than half of mothers (60.3%) reported that their child used a pacifier. However, only 43.9% of respondents were aware that pacifier use could reduce the risk of SIDS.

Despite a significant decline in SIDS-related deaths, infant mortality due to SIDS remains high. Ongoing awareness campaigns are essential to reducing risk and improving infant care among both parents and healthcare professionals. Caregiver education should focus on modifying habits and shaping health-conscious behaviors. Meanwhile, healthcare workers should be responsible for delivering consistent and unified information [24,39,40]. According to Coll et al. [24], it is also important to identify population groups that may require targeted education or support.

A limitation of the study was that the sample included only a selected part of Poland, which limits the representativeness of the results. It would be necessary to conduct research on a larger scale to obtain a more comprehensive picture. Additionally, including both legal guardians as study participants would allow for a more accurate assessment of the level of knowledge regarding safe infant sleep.

An advantage of the article is its focus on the important and so far insufficiently studied issue of parents’ knowledge about safe infant sleep, which is a significant global health concern. The analysis within the Polish context represents a valuable contribution to both the national and international scientific literature.

## 5. Conclusions

Knowledge of safe sleep recommendations, including sleep environment, sleep position, and spatial organization, does not always translate into proper care practices. Strengthening this awareness can help reduce the current mortality rates associated with SIDS and SUID. Understanding the motivations of caregivers and how they perceive medical information is crucial for effective prevention of infant sleep safety. Similarly, identifying trends in contemporary infant care practices is critical. Further research is needed to evaluate the effectiveness of educational programs to increase knowledge, skills, and competencies related to infant care in the first year of life.

## Figures and Tables

**Table 1:** Characteristics of the study group (*n* = 451).

Characteristics	*n*	%
Age: below 25 years	43	10.2
Age: 26–30 years	197	43
Age: above 30 years	208	46.1
Primary education	2	0.4
Secondary education	72	16
Higher education:	377	83.6
Medical occupation	107	23.7
Non-medical occupation	344	76.3
natural birth	93	93
stimulated/induced birth	162	162
cesarean section	196	196
One child	268	59.4
Two children	149	33
Three children	32	7.1
Four children	2	0.4

**Table 2:** Frequency of correct and incorrect answers on respective questions of questionnaire regarding safe sleep knowledge (*n* = 451).

Questions and Correct Answers (True or False)	Correct Answers		Incorrect Answers	
	*n*	%	*n*	%
Infants should be placed on their stomachs if awake and supervised: True	410	90.9	41	9.1
Prone sleep position is safe for infants: False	311	69.0	140	31.0
Side sleep position is safe for infants: False	184	40.8	267	59.2
Supine sleep position is safe for infants: True	401	88.9	50	11.1
Co-sleeping with parents or siblings is safe for infants: False	336	74.5	115	25.5
Infants should sleep separately in their own crib: True	353	78.3	98	21.7
Objects (pillows, toys, blankets) can be placed in the crib: False	444	98.4	7	1.5
Swaddling (tight wrapping, use of swaddles) extends sleep duration: True	237	52.5	214	47.5
Swaddling can disrupt hip joint development: True	269	59.6	182	40.4
Breastfeeding should start within the first hour after birth: True	409	90.7	44	9.8
Breastfeeding mothers should be on an elimination diet: False	16	3.5	435	96.5
Feeding on demand is recommended in the first months: True	48	10.6	403	89.4
Using a pacifier during breastfeeding initiation can disrupt sucking and lactation: True	387	85.8	64	14.2
Pacifier use can reduce the risk of SIDS: True	198	43.9	253	56.1

**Table 3:** Sleeping location depending on child’s age.

Child’s Age	Sleeping Place	Number of Responses	Responses (%)	Respondents (%) *
0–3 months (*n* = 103)	Crib	89	47.3	86.4
	Sleeping basket	4	2.1	3.9
	Bassinet	7	3.7	6.8
	Stroller	24	12.8	23.3
	Nest	6	3.2	5.8
	In parents’ bed	34	18.1	33.0
	Lying on mom/dad	24	12.8	23.3
4–7 months (*n* = 167)	Crib	128	46.9	76.6
	Sleeping basket	8	2.9	4.8
	Bassinet	7	2.6	4.2
	Stroller	27	9.9	16.2
	Nest	7	2.6	4.2
	In parents’ bed	76	27.8	45.5
	Lying on mom/dad	20	7.3	12.0
8–12 months (*n* = 100)	Crib	70	41.9	70.7
	Sleeping basket	1	0.6	1.0
	Bassinet	3	1.8	3.0
	Stroller	13	7.8	13.1
	Nest	4	2.4	4.0
	In parents’ bed	63	37.7	63.6
	Lying on mom/dad	13	7.8	13.1
13–18 months (*n* = 81)	Crib	58	45.3	72.5
	Sleeping basket	3	2.3	3.8
	Bassinet	2	1.6	2.5
	Stroller	11	8.6	13.8
	Nest	4	3.1	5.0
	In parents’ bed	39	30.5	48.8
	Lying on mom/dad	11	8.6	13.8

* Multiple-choice question (does not sum to 100%).

**Table 4:** Sleeping positions used in the study group.

Sleeping Position	Number of Responses	Responses (%)	Respondents (%) *
Side sleep position	169	29.3	37.5
Prone sleep position	54	9.4	12.0
Supine sleep position	354	64.1	78.5

* Multiple-choice question (does not sum to 100%).

**Table 5:** Sleeping positions by child’s age.

Age Group	Sleeping Position	Number of Responses	Responses (%)	Respondents (%) *
0–3 months (*n* = 103)	Side sleep position	42	30.2	40.8
	Prone sleep position	14	10.1	13.6
	Supine sleep position	83	59.7	80.6
4–7 months (*n* = 167)	Side sleep position	51	23.5	30.5
	Prone sleep position	20	9.2	12.0
	Supine sleep position	146	67.3	87.4
8–12 months (*n* = 100)	Side sleep position	42	32.3	42.0
	Prone sleep position	13	10.0	13.0
	Supine sleep position	75	57.7	75.0
13–18 months (*n* = 81)	Side sleep position	34	37.4	42.0
	Prone sleep position	7	7.7	8.6
	Supine sleep position	50	54.9	61.7

* Multiple-choice question (does not sum to 100%).

## Data Availability

The authors have reviewed and edited the output and take full responsibility for the content of this publication.

## Abbreviations

The following abbreviations are used in this manuscript:

SUID	sudden unexpected infant death
SISD	sudden infant death syndrome
AAP	American Academy of Pediatrics
